# Severe *Plasmodium falciparum* Malaria Is Associated with Circulating Ultra-Large von Willebrand Multimers and ADAMTS13 Inhibition

**DOI:** 10.1371/journal.ppat.1000349

**Published:** 2009-03-20

**Authors:** Deirdre Larkin, Bas de Laat, P. Vince Jenkins, James Bunn, Alister G. Craig, Virginie Terraube, Roger J. S. Preston, Cynthia Donkor, George E. Grau, Jan A. van Mourik, James S. O'Donnell

**Affiliations:** 1 Haemostasis Research Group, Trinity Centre for Health Sciences, Trinity College Dublin, Dublin, Ireland; 2 Sanquin, Amsterdam, The Netherlands; 3 Departments of Community Health and Paediatrics, College of Medicine, Blantyre, Malawi; 4 School of Tropical Medicine, Liverpool, United Kingdom; 5 Komfo Anokye Teaching Hospital, Kumasi, Ghana; 6 Department of Pathology, University of Sydney, Sydney, New South Wales, Australia; 7 National Centre for Hereditary Coagulation Disorders, St James's Hospital, Dublin, Ireland; INSERM, France

## Abstract

*Plasmodium falciparum* infection results in adhesion of infected erythrocytes to blood vessel endothelium, and acute endothelial cell activation, together with sequestration of platelets and leucocytes. We have previously shown that patients with severe infection or fulminant cerebral malaria have significantly increased circulatory levels of the adhesive glycoprotein von Willebrand factor (VWF) and its propeptide, both of which are indices of endothelial cell activation. In this prospective study of patients from Ghana with severe (n = 20) and cerebral (n = 13) *P. falciparum* malaria, we demonstrate that increased plasma VWF antigen (VWF∶Ag) level is associated with disproportionately increased VWF function. VWF collagen binding (VWF∶CB) was significantly increased in patients with cerebral malaria and severe malaria (medians 7.6 and 7.0 IU/ml versus 1.9 IU/ml; p<0.005). This increased VWF∶CB correlated with the presence of abnormal ultra-large VWF multimers in patient rather than control plasmas. Concomitant with the increase in VWF∶Ag and VWF∶CB was a significant persistent reduction in the activity of the VWF-specific cleaving protease ADAMTS13 (∼55% of normal; p<0.005). Mixing studies were performed using *P. falciparum* patient plasma and normal pooled plasma, in the presence or absence of exogenous recombinant ADAMTS13. These studies demonstrated that in malarial plasma, ADAMTS13 function was persistently inhibited in a time-dependent manner. Furthermore, this inhibitory effect was not associated with the presence of known inhibitors of ADAMTS13 enzymatic function (interleukin-6, free haemoglobin, factor VIII or thrombospondin-1). These novel findings suggest that severe *P. falciparum* infection is associated with acute endothelial cell activation, abnormal circulating ULVWF multimers, and a significant reduction in plasma ADAMTS13 function which is mediated at least in part by an unidentified inhibitor.

## Author Summary

Malaria is caused by infection of red blood cells (erythrocytes) with protozoan parasites of the genus *Plasmodium*. Infected erythrocytes adhere to and disrupt the inner lining, or endothelium, of small blood vessels, especially those of the brain, resulting in blockage and subsequent cerebral malaria. We have studied the effect of *Plasmodium falciparum* infection on the endothelial cell activation marker, the multimeric adhesive protein von Willebrand factor (VWF) in a cohort of patients with severe infection or cerebral malaria. We demonstrate that malarial infection in these patients is associated with abnormally high levels of ultra-large VWF in blood plasma, and that VWF functional ability as measured by collagen binding is disproportionately increased as compared to normal plasmas. Circulating levels of the VWF-specific cleaving enzyme ADAMTS13 is reduced to ∼55% of normal in patients, and plasma mixing studies demonstrate the presence of an inhibitor of ADAMTS13 function. Thus, severe *P. falciparum* infection results in disruption of the endothelium, causing release of ultra-large VWF. Together with reduced ADAMTS13 levels, and an unidentified inhibitor of ADAMTS13, this may contribute to the pathophysiology of malaria.

## Introduction

In spite of the significant mortality associated with *P. falciparum* infection, the molecular mechanisms involved in its pathophysiology remain poorly understood. However, sequestration of *P. falciparum*-infected erythrocytes (IE) in the microvasculature of vital organs including the brain and placenta plays a key role in this process [1]. Previous studies have demonstrated that sequestration involves adhesion of IE to endothelial cell (EC) surfaces. This process is mediated by various parasite-related ligands, including *P. falciparum* erythrocyte membrane protein 1 (PfEMP1), expressed on the surface of IE [2]. Furthermore, a number of specific receptors expressed on EC surfaces are important in regulating IE adhesion, including thrombomodulin, CD36, thrombospondin, intercellular adhesion molecule-1 (ICAM-1), vascular adhesion molecule-1 (VCAM-1), P-selectin and E-selectin. Expression of these receptors varies significantly between different vascular beds, and can be regulated in response to inflammatory cytokines (e.g. TNF and interleukin-1) [3],[4]. Consequently, EC activation plays a critical role in regulating IE cytoadherence [5].

Von Willebrand factor (VWF) is a large plasma glycoprotein that plays a critical role in primary haemostasis by mediating the adhesion of platelets to sites of vascular injury [6]. *In vivo* VWF biosynthesis is limited to EC and megakaryocytes [7]. VWF synthesised within EC is either constitutively secreted into the plasma, or alternatively stored within specific intracellular organelles known as Weibel-Palade (WP) bodies [8]. Following EC activation by a variety of secretagogues including thrombin, fibrin and histamine, VWF and its propeptide are secreted in equimolar concentrations from the WP bodies [9]. We recently reported marked increased plasma VWF and VWF propeptide levels in severe *P. falciparum* infection, consistent with acute EC activation [10]. Indeed, children with cerebral malaria (CM) had VWF propeptide levels exceeding those typically observed in fulminant vascular diseases such as thrombotic thrombocytopenic purpura (TTP) [11]. Subsequently, a study of 14 healthy volunteers infected with *P. falciparum* showed that the increased plasma VWF and VWF propeptide levels develop soon after the onset of blood stage infection [12]. Consequently, acute EC activation constitutes an early feature of *P. falciparum* malaria infection, and may therefore be important in the pathogenesis of progression to severe or cerebral malaria respectively.

Plasma VWF plays a critical role in primary haemostasis by mediating the adhesion of platelets to sites of vascular injury [6],[8]. Following endothelial disruption, VWF binds to exposed collagen in the subendothelial matrix. This anchored VWF undergoes marked conformational changes in response to shear stress exerted by the circulating blood, and can then tether platelets through specific binding of the platelet Gp Ib-IX-V receptor [9],[13]. Accumulating evidence suggests that platelet adhesion and aggregation may play important roles in facilitating cytoadhesion of *P. falciparum* IE to activated EC [14]–[16]. However, it remains unclear if the increased plasma VWF levels play any direct role in mediating this process, or whether they merely serve as a marker of acute EC activation. Nevertheless, elegant studies using a novel llama-derived nanobody have demonstrated that a significant proportion of circulating plasma VWF in *P. falciparum* infected patients is present in an active platelet GpIb-binding conformation [12]. Furthermore, plasma VWF∶Ag levels in patients with malaria inversely correlate with platelet count [12], and we have previously shown that plasma VWF propeptide levels correlate with other established biochemical markers of malaria severity, including plasma lactate [10].

To further elucidate the mechanism responsible for quantitative and qualitative variations in plasma VWF levels in malaria, we collected plasma samples from a cohort of children with laboratory confirmed severe *P. falciparum* infection, or full-blown cerebral malaria. We demonstrate herein that severe *P. falciparum* malaria is associated not only with increased plasma VWF antigen (VWF∶Ag) levels, but an even more marked increase in VWF activity as determined by collagen binding assay (VWF∶CB), due to the presence of abnormal circulating ultra-large VWF (ULVWF) multimers. In addition, we also demonstrate that the presence of ULVWF is associated with a significant reduction in plasma levels of the VWF cleaving protease ADAMTS13 (A Disintegrin And Metalloproteinase with ThromboSpondin type-1 repeats), and an unidentified inhibitor of ADAMTS13 activity present in the plasma of children with severe *P. falciparum*.

## Methods

### Patients

Patients were recruited from those presenting with severe malaria to the Komfo Anokye Teaching Hospital in Kumasi, Ghana, as previously described [10]. Subjects were children aged between 6 months and 6 years, recruited after written informed consent had been obtained. Ethical approval was granted by the committee on human research, publications and ethics (CHRPE), School of Medical Sciences, University of Science and Technology, Kumasi, and also by the Liverpool School of Tropical Medicine research ethics committee. For each subject, clinical details were obtained at presentation, and *P. falciparum* infection confirmed on thick blood films. Venous blood samples were collected, before standard anti-malarial treatment was commenced in all patients.

Cerebral malaria was defined as a Blantyre coma score of two or less in a child with malarial parasitaemia, and without any other cause of coma (e.g. hypoglycaemia or meningitis) [17]. For the cohort of children with cerebral malaria (n = 13), follow-up samples were also collected at 24 and 72 hours respectively, following admission and after commencement of treatment. Non-cerebral severe malaria was defined according to standard World Health Organization criteria (WHO, 2000), which included severe anaemia (<5 g/dl), prostration, convulsions, and respiratory distress (n = 20), but which did not meet the criteria for cerebral malaria. Finally, healthy controls (n = 25) were recruited from children attending for immunisation, surgery, or outpatient surgical review.

### VWF and ADAMTS13 antigen and activity assays

From each patient and control, 1.2 ml of venous blood was collected into 3.2% citrate (1∶9 vol/vol), and immediately placed on ice. After centrifugation at 3000 *g* for 20 min at 4°C, plasma aliquots were stored at −80°C. Plasma VWF∶Ag levels were performed as previously described [10],[18]. VWF∶CB levels were determined using a commercial ELISA method (Technoclone, UK) as before [19]. VWF multimer analysis was performed according to Ruggeri *et al* with minor modifications [20]. Sodium dodecyl sulfate (SDS)-agarose gel electrophoresis was performed using 1.5% agarose gels, and VWF multimer composition, visualised using HRP-labelled polyclonal rabbit anti-human VWF (Dako, Glostrup, Denmark). For objectively quantifying differences in VWF multimer composition, densitometry was performed using ImageJ software (Image Processing and Analysis in Java). ADAMTS13 activity was determined by FRETS-VWF73 assay (Peptides International, Kentucky, USA), and ADAMTS13 antigen levels by ELISA using murine monoclonal antibodies (kind gift of Dr H. Feys, Washington University, Saint Louis, USA) as previously reported [21].

### ADAMTS13 inhibitor studies

To investigate ADAMTS13 inhibition, individual malaria plasmas (n = 4) were mixed in different proportions (25%∶75% or 50%∶50%) with pooled normal plasma. ADAMTS13 activity using the FRETS-VWF73 assay (Peptides International, Kentucky, USA), was assessed immediately following mixing, and after incubation at 37°C for 15 min or 30 min respectively. Recombinant human ADAMTS13 (cDNA kind gift of Dr R. Montgomery, Medical College of Wisconsin, USA) was stably expressed in HEK293 cells, and purified as previously described [18]. Recombinant ADAMTS13 was adjusted to normal pooled plasma activity level (1 U/ml) and then added to *P. falciparum*-infected (n = 4), or control plasma samples (n = 4), and ADAMTS13 activity timecourse assessed as above (FRETS-VWF73 assay). Finally, plasma concentrations of IL-6 (Abcam, Cambridge, UK) and TSP-1 (R&D systems, Minneapolis, USA) were measured using commercial ELISA kits, in accordance with the manufacturer's instructions, and plasma haemoglobin levels were measured using a Sysmex XE 5000 analyser. No red cells were detected in the platelet poor plasma preparations.

### Statistical analysis

All statistical analyses were performed using the SPSS statistics package (version 4.02, SPSS Inc), and statistical significance was assigned at a value of p<0.05. Normally distributed data are presented as mean +/− SEM, and differences between patients and controls analyzed using the two sample Student's t test. For nonparametric data, medians and ranges were calculated and nonparametric tests for statistical significance were performed using Mann-Whitney test.

## Results

### Severe *P. falciparum* malaria influences plasma VWF multimer composition

**Figure 1:**
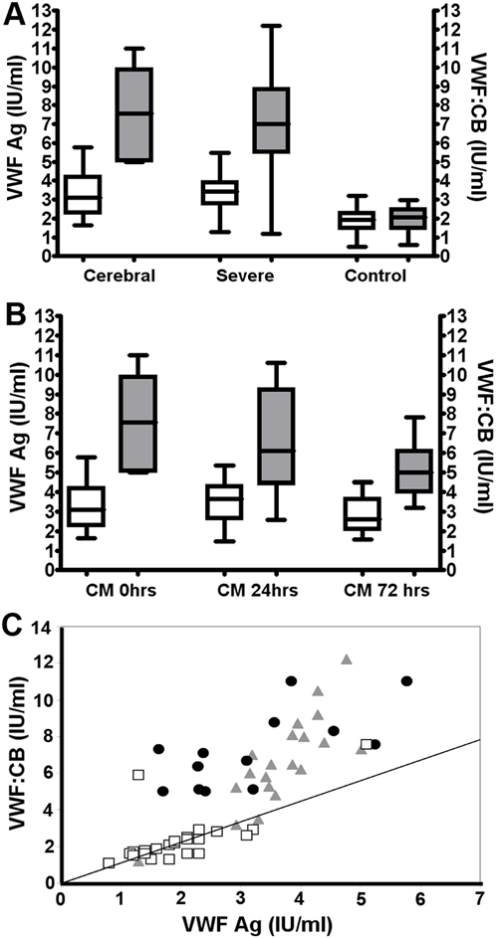
Severe *Plasmodium falciparum* malaria influences plasma VWF antigen level and collagen binding activity. (A) Plasma VWF∶Ag levels (*white bars – left Y axis*) were measured by ELISA, and VWF activity by collagen binding activity (VWF∶CB) (*grey bars – right Y axis*). Each plasma sample was tested in duplicate at three dilutions, and median values for each group are shown. VWF∶Ag and VWF∶CB levels were markedly elevated in patients with cerebral malaria and in children with severe malaria at presentation compared to levels in healthy control children. (B) In a cohort of children with cerebral malaria (CM), the time-course of VWF∶Ag and VWF∶CB levels following admission and commencement of anti-malarial therapy was assessed using follow-up plasma samples collected after 24 and 72 hours respectively. (C) Although both VWF∶Ag and VWF∶CB were increased in all cases of *P. falciparum* malaria, the relative increase observed in plasma VWF∶CB levels was significantly higher (p<0.05), such that the ratio of CB to Ag was consistently >1 in children with CM (n = 13; •) or SM (n = 20; ▴) at presentation compared to healthy control subjects (n = 25; □). (*Hashed line indicates 1∶1 ratio*).

In children with cerebral (CM) and non-cerebral severe (SM) *P. falciparum* malaria, plasma VWF∶Ag levels were significantly elevated at presentation (medians 3.1 and 3.4 IU/ml; p<0.05) (Fig 1A). In addition, plasma VWF activity levels (VWF∶CB) were also markedly increased (medians 7.6 and 7.0 IU/ml; p<0.05). In the CM cohort, plasma VWF∶Ag levels remained elevated during the 72 hours following admission (Fig 1B). In contrast, VWF∶CB activity decreased progressively over this same time period (Time 0 hrs – 7.6 IU/ml; 24 hrs – 6.1 IU/ml; 72 hrs – 5.0 IU/ml respectively, p = 0.03), in a manner similar to that previously reported for VWF propeptide levels [10]. Although plasma VWF∶Ag and VWF∶CB were both increased in children with CM or SM at initial presentation, the observed increase in VWF∶CB was much greater compared to that in plasma VWF∶Ag levels, so that the ratio of CB∶Ag was consistently increased compared to that observed in healthy control children (Fig 1C). Moreover, in the cohort of children with CM, despite the progressive reduction in plasma VWF∶CB activity following admission to hospital and commencement of anti-malarial therapy (Fig 1B), the ratio of VWF∶Ag to VWF∶CB still remained skewed even 72 hours after initiation of anti-malarial therapy (data not shown).

**Figure 2:**
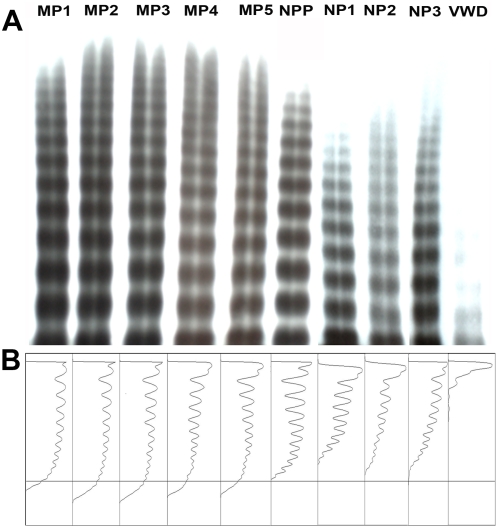
Cerebral, and non-cerebral severe *Plasmodium falciparum* malaria, are associated with abnormal high molecular weight VWF multimers. (A) Plasma VWF multimer distribution was analysed by non-reducing agarose gel electrophoresis. Plasma from children with severe *P. falciparum* (MP1, MP2, MP3, MP4, MP5) demonstrated abnormal HMW-VWF multimers which were not observed in normal pooled plasma (NPP), nor in healthy control children (NP1, NP2, NP3). Plasma from a patient with known type 2A von Willebrand disease (with characteristic loss of multimers) has been included as negative control (VWD). (B) Densitometric scans of the lanes in (A). Individual panels correspond with individual lanes. The horizontal axis is optical density. Abnormal HMW multimers are present in MP1 to MP5 (highlighted by bands extending beyond arbitrary line) but not apparent in NPP or healthy controls (NP1 to NP3).

It is well-established that the VWF∶CB is particularly sensitive to circulating high molecular weight multimer (HMWM) VWF [22]. VWF multimer analysis and densitometry confirmed that abnormal ultra-large (UL) VWF multimers were present in the plasma of children with either CM or SM respectively (Fig 2). Furthermore, these abnormal multimers were not observed in the plasma of normal Ghanaian children. Cumulatively, these data further support the hypothesis that acute endothelial cell activation constitutes a hallmark of severe *P. falciparum* infection, but also confirm the presence of abnormal circulating ULVWF multimers in malarial plasma.

### Plasma ADAMTS13 antigen and activity in severe *P. falciparum* malaria

ADAMTS13 regulates normal plasma VWF multimer composition and thereby activity, through cleavage at the Tyr 1605- Met 1606 bond within the VWF A2 domain [9],[23]. To determine the molecular mechanism(s) responsible for the markedly increased VWF∶CB and circulating ULVWF in severe malaria infection, we investigated plasma ADAMTS13 levels in children with CM, SM and normal healthy controls.

Although plasma ADAMTS13 antigen and activity levels were previously shown to be markedly reduced in neonates [21], normal ADAMTS13 levels have not previously been described for older African children. In our normal control children (minimum age 6 months), median ADAMTS13 activity levels were 1.07 U/ml (children aged 6–12 months; n = 10) and 1.23 U/ml (children 1–6 years; n = 15). These levels were not significantly different (p = 0.45), and fell well within our normal adult plasma ADAMTS13 activity range. In addition, plasma ADAMTS13 activity levels were not significantly influenced by gender. These findings are consistent with another recent study that determined plasma ADAMTS13 levels in a cohort of U.K. children [24].

**Figure 3:**
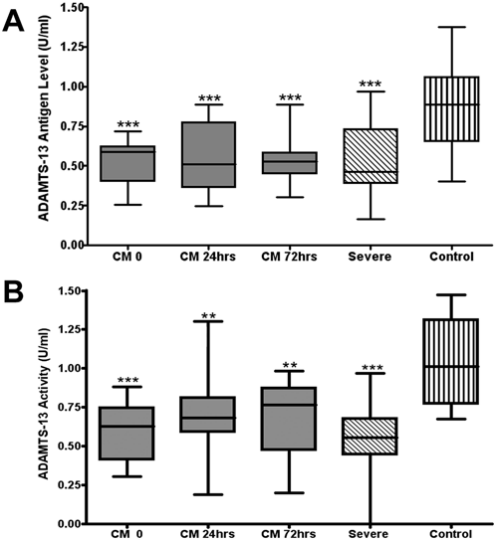
Effect of severe *P. falciparum* malaria infection on plasma ADAMTS13 antigen and activity. (A) Plasma ADAMTS13 antigen levels were significantly reduced in children with CM (median = 0.6 U/ml; p<0.005; Mann Whitney) and SM (median = 0.55 U/ml; p<0.005) at presentation compared to healthy control children. Furthermore, in the CM cohort, ADAMTS13 antigen levels remained significantly reduced at 24 hrs and 72 hrs following commencement of anti-malarial therapy. (* *p*<0.05; ** *p*<0.005; *** p<0.0005). (B) Plasma ADAMTS13 activity levels, determined by FRETS-VWF73 assay, were also significantly reduced in cases of both CM (median = 0.63 U/ml; p<0.005: Mann Whitney) and SM (median = 0.56 U/ml; p<0.005) compared to healthy controls.

ADAMTS13 antigen levels were significantly lower in children with severe malaria at presentation than in controls (Fig 3A). Similarly, plasma ADAMTS13 activity levels were also significantly reduced in both CM (median = 0.63 U/ml; p<0.001; Mann Whitney) and SM (median = 0.56 U/ml; p<0.001) at initial presentation (Fig 2B). Furthermore, in the cohort of children with CM, ADAMTS13 activity remained significantly reduced during the subsequent 72 hour follow-up period. Although ADAMTS13 antigen and ADAMTS13 activity levels were reduced to comparable degrees in most children with SM and CM, four patients were identified with reduced activity∶antigen ratios (<0.7). These novel data are consistent with a recent report of acquired ADAMTS13 deficiency in paediatric patients with other causes of severe sepsis [25], but contrast with the previous findings of de Mast *et al*, who reported normal plasma ADAMTS13 activity by FRETS-VWF73 assay during the early stages of *P. falciparum* infection [12].

### Inhibition of ADAMTS13 activity in severe *P. falciparum* plasma

**Figure 4:**
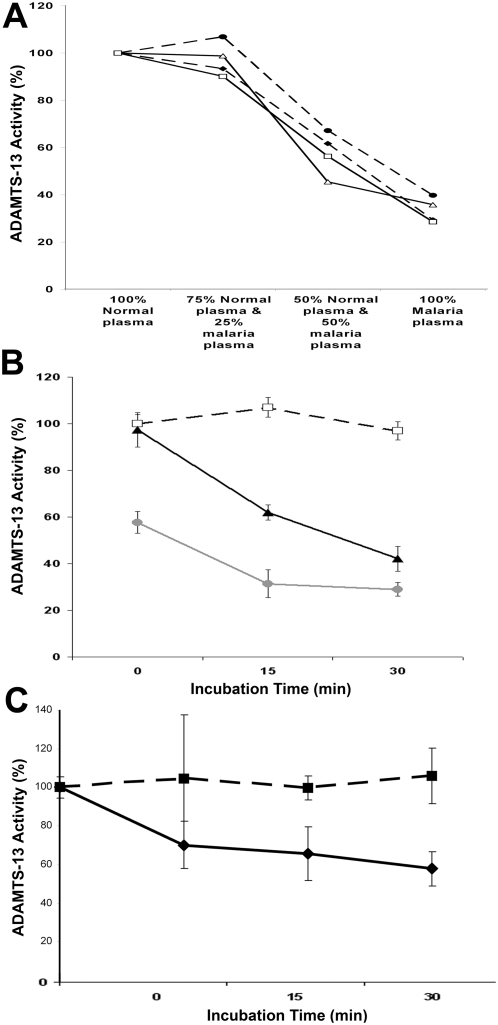
Plasma ADAMTS13 activity inhibition in *P. falciparum* malaria. (A&B) To investigate further the mechanisms responsible for the marked and discrepant increase in plasma VWF∶CB, and the significant reduction in ADAMTS13 activity, we investigated the effects of mixing malaria plasma with normal plasma. Plasma from four different children (□, •, ▵, ⧫) with SM (each with baseline ADAMTS13 activities of ∼0.4 U/dl) were mixed in various proportions with pooled normal plasma, and ADAMTS13 activity determined. No evidence of an immediate ADAMTS13 inhibitor effect was observed (Fig 4A). However following incubation at 37°C for 15 min or 30 min (Fig 4B), significant ADAMTS13 inhibition was observed in malaria plasmas at either 75%∶25% (▴), or 50%∶50% (•), but not in normal control plasma (□). All results represent mean±SEM. (C) To further investigate whether malarial plasma contained an ADAMTS13 inhibitor, individual malaria plasma samples (n = 4) and control plasmas (n = 4) were spiked with recombinant human ADAMTS13. Again, significant inhibition of rADAMTS13 activity (means±SEM) was observed only in malaria plasma (⧫) but not in normal plasma (▪).

To investigate whether the reduction in ADAMTS13 activity in malaria plasma might be attributable to the presence of an inhibitor, we performed mixing studies of malaria and normal plasma respectively. Following immediate mixing, no ADAMTS13 inhibition was apparent (Fig 4A). However following incubation at 37°C, clear evidence of a time-dependent inhibitory effect was observed (Fig 3B). Indeed, ADAMTS13 activity in normal plasma was reduced by approximately 60% after pooled normal plasma was incubated in a 75%∶25% mix with malarial plasma for 30 mins. Similar levels of inhibition were observed using four different malaria plasma samples, but not after similar incubation with normal control plasmas. This ability of malaria plasma to directly inhibit ADAMTS13 activity was confirmed by spiking malarial plasmas with recombinant human ADAMTS13 (Fig 3C). Once again, significant time-dependent inhibition of FRETS-VWF73 activity was observed in the plasmas of children with severe *P. falciparum* (p<0.001), but not in normal control plasmas.

### Effects of severe *P. falciparum* malaria on ADAMTS13 inhibitors in plasma

Proteins suggested to regulate the ability of plasma ADAMTS13 to cleave full-length VWF include interleukin 6 (IL-6) [26], thrombospondin-1 (TSP-1) [27], thrombin and plasmin [19], free plasma haemoglobin [28], and most recently reduced factor VIII (FVIII) levels [29]. We found that plasma IL-6 levels were significantly elevated at presentation in children with either CM (mean 240 pg/ml; p<0.001; Student's t-test) or SM (mean 217 pg/ml; p = 0.01) compared to normal controls (Fig 4A). However, although plasma IL-6 levels were increased in these children, the levels did not approach those previously reported necessary to inhibit ADAMTS13 activity [26]. Furthermore, in spiking experiments we found no inhibitory effect of IL-6 (final concentration range 0.01–10 µg/ml) in a static ADAMTS13 activity assay (Fig 4B). Disseminated intravascular coagulopathy (DIC) is associated with enhanced thrombin generation, and consumption of circulating FVIII, both of which can inhibit VWF proteolysis by ADAMTS13 [19],[29]. However, in children with CM or SM respectively, we observed significantly increased plasma FVIII∶C levels (Fig 4C), confirming previous reports suggesting that fulminant DIC is a rare complication of severe malaria [30],[31]. Intravascular haemolysis is a recognised complication of malarial infection. Moreover, increased plasma haemoglobin has previously been described to inhibit ADAMTS13 activity [28],[32]. In children with CM or SM, we observed only minor increased plasma haemoglobin concentrations (Fig 4D), well below that previously described to significantly inhibit ADAMTS13 activity [28]. Finally, in contrast to the increased plasma levels of IL-6 and FVIII∶C, we found that plasma TSP-1 levels were not significantly elevated in children with either CM or SM compared to pooled normal plasma (data not shown).

## Discussion

VWF undergoes complex post-translational modification within EC prior to secretion, including two distinct polymerization steps [7]. Dimerisation occurs within the ER, through the formation of inter-subunit C-terminal disulphide bonds. Subsequently in the Golgi, VWF dimers are formed into multimers through another round of N-terminal disulphide bond formation. Consequently, VWF is constitutively secreted into the plasma as multimers of varying size [7],[9]. In contrast, VWF stored within WP bodies exists predominantly as ULVWF multimers that are released into plasma in response to EC activation [33],[34]. The multimeric composition of VWF is a critical determinant of its functional activity. Larger VWF multimers bind collagen and activated platelets with ∼100 fold higher affinities compared to monomers, and are thus more efficient in inducing platelet aggregation [9],[13]. In this study, we demonstrate that severe *P. falciparum* malaria is associated with an accumulation of hyper reactive ULVWF multimers in the plasma, and thereby a marked increase in plasma VWF activity (Fig 1). The mechanism(s) responsible for this accumulation of abnormal ULVWF remains unclear. However we recently demonstrated that severe *P. falciparum* malaria results in fulminant, acute EC activation together with marked exocytosis of WP bodies, which are responsible for the majority of the increase in plasma VWF levels [10].

Following secretion, ULVWF multimers released from WP bodies normally undergo rapid, partial proteolysis on the endothelial cell surface by a VWF-specific cleaving protease termed ADAMTS13 [35],[36]. ADAMTS13 cleaves the Y1605/M1606 peptide bond in the VWF A2 domain, thereby preventing the accumulation of ULVWF multimers in normal plasma, and thus also regulating VWF functional activity [23],[36]. However, it remains largely unclear how VWF proteolysis by ADAMTS13 is regulated *in-vivo*, although recent studies have identified some putative mechanisms of cofactor enhancement [29] and inhibition [19] respectively. In this study, we demonstrate that plasma ADAMT13 antigen and activity levels are both significantly reduced in children with CM or SM at presentation (Fig 3). This finding differs from early *P. falciparum* malaria, where plasma ADAMTS13 levels were previously reported to be within the normal range [12]. Decreased plasma ADAMTS13 antigen level may be partly attributable to a reduction in hepatic ADAMTS13 synthesis. However, increased consumption of ADAMTS13 in the setting of sustained, systemic release of ULVWF has also been previously described [37]. Interestingly, ADAMTS13 antigen and activity levels did not improve significantly during the 72 hours following commencement of anti-malarial therapy in children with CM (Fig 3). In contrast, we observed a significant fall in VWF collagen binding activity (and VWF propeptide) over this time period, but not in plasma VWF antigen levels. Cumulatively, these data suggest that acute EC activation and ongoing WP body secretion are essential in order to maintain circulating ULVWF in *P. falciparum* malaria.

Although ADAMTS13 antigen and activity were both significantly reduced in children with severe *P. falciparum malaria* compared to normal controls, absolute plasma ADAMTS13 levels remained above 50% (median 0.63 U/ml or 63%). These novel data are in keeping with those of Nguyen *et al.*, who reported a similar reduction in ADAMTS13 activity (mean 57.4%) in children with non-malarial severe sepsis [25]. Furthermore, Sosothikul *et al* also observed comparable reductions in plasma ADAMTS13 activity in a cohort of paediatric patients with Dengue virus [38]. The correlation between these respective data is noteworthy, given that different methods (FRETS-VWF75; full-length VWF cleavage; and flow chamber assay) were used in the three studies to determine plasma ADAMTS13 activity. Nevertheless, whether this modest reduction in plasma ADAMTS13 plays an important role in mediating the ULVWF accumulation in severe malaria remains unclear. Previous studies have suggested that ADAMTS13 activity levels above 10% are sufficient to maintain normal plasma VWF multimer composition, at least in the absence of any associated acute EC activation [39]. Consequently, it seems likely that *in-vivo* inhibition of plasma ADAMTS13 activity may also be occurring in children with CM or SM respectively.

To further investigate the mechanism(s) responsible for the persistence of ULVWF in the presence of reduced but significant residual ADAMTS13, we investigated ADAMTS13 activity inhibition in malarial plasma. Using a FRETS-VWF73 assay to quantify residual ADAMTS13 activity, moderate time-dependent inhibition of the ADAMTS13 activity in normal pooled plasma was observed following incubation with an equal volume of malarial plasma. Furthermore, similar inhibition was clearly apparent when recombinant human ADAMTS13 was spiked into malarial plasma, but not into normal plasma (Fig 3). Cumulatively, these findings support the hypothesis that severe *P. falciparum* plasma may contain an inhibitor of ADAMTS13 activity. However, current *in vitro* ADAMTS13 assays are performed under non-physiological conditions (low ionic strength buffer containing barium and urea). Consequently, it is difficult to reliably extrapolate *in vitro* results to VWF processing *in vivo*, and consequent translational significance.

**Figure 5:**
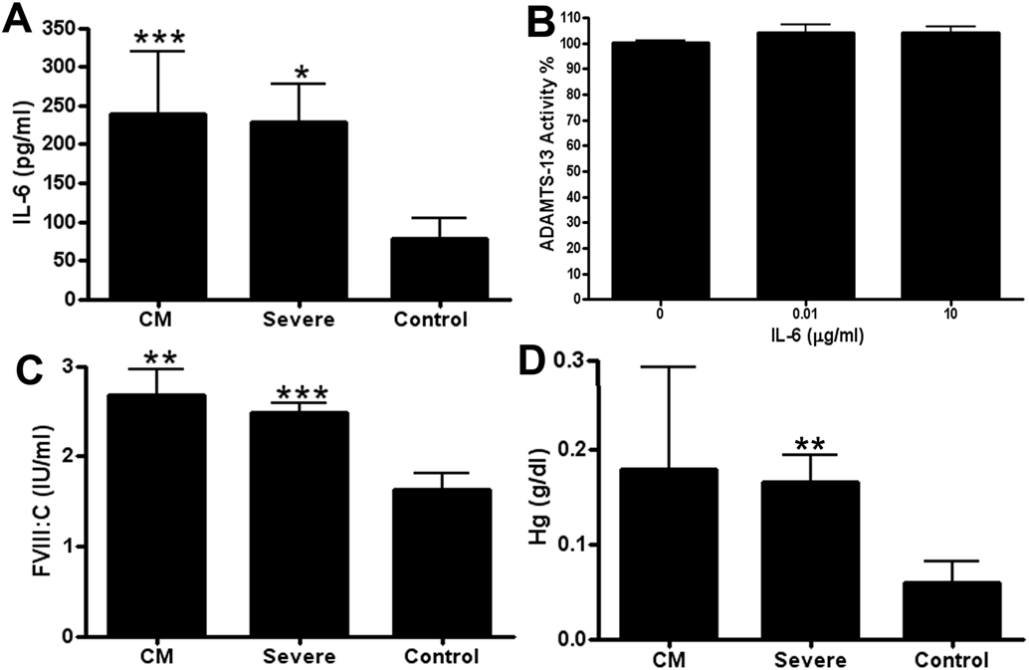
Severe *P. falciparum* malaria and plasma ADAMTS13 inhibitor levels. (A&B) In children with CM or SM, plasma IL-6 levels (means±SEM) were significantly increased compared to those in normal controls. However the absolute concentrations of IL-6 did not reach that previously described as being required to inhibit ADAMTS proteolysis of VWF, and in spiking experiments we found no inhibitory effect of IL-6 (final concentration range 0.01–10 µg/ml) on ADAMTS13 activity in pooled normal plasma (FRETS-VEF73 assay). (C) Reduced plasma FVIII levels have recently been reported to inhibit ADAMTS13 activity but plasma FVIII∶C levels (means±SEM) were significantly increased in children with severe *P. falciparum* malaria. (D) Although, intravascular haemolysis is a recognised complication of malarial infection, and free plasma haemoglobin has been shown to inhibit ADAMTS13 activity, only minor increased plasma haemoglobin concentrations were observed in children with CM or SM, well below that previously described to significantly inhibit ADAMTS13 activity. (* *p*<0.05; ** *p*<0.005; *** p<0.0005).

Although the mechanisms underlying the physiological regulation of ADAMTS13 enzymatic activity are not well-defined, previous studies have identified several putative inhibitors, including IL-6, TSP-1, thrombin, free plasma haemoglobin, and reduced FVIII (FVIII∶C) levels [19], [26]–[29],[39]. In keeping with previous reports, we observed significantly elevated plasma IL-6 levels in children with both CM and SM respectively (Fig 5) [40],[41]. Plasma haemoglobin levels were also slightly increased in both groups of children. However, the absolute plasma concentrations of both IL-6 and haemoglobin were well below those previously reported to significantly ADAMTS13 activity *in-vitro* [26],[28]. Whether these inhibitors might interact synergistically, or indeed whether their true *in-vivo* inhibitory capacity is accurately reflected in an *ex-vivo* ADAMTS13 functional assay remains unclear [39]. Finally, and again in keeping with previous studies, consumption of coagulation factor VIII (a recently described ADAMST13 cofactor [29]) was not a feature of severe *P. falciparum* malaria. Thus the mechanism responsible for ADAMTS13 inhibition in malarial plasma remains unknown, and cannot be explained by quantitative variation in any of the previously reported plasma ADAMTS13 inhibitors.

In conclusion, based upon our findings we propose that the presence of hyper-reactive ULVWF multimers in the plasma of children with severe *P. falciparum* malaria is the result of (i) acute EC activation and release of ULVWF from WP bodies; (ii) significantly reduced plasma ADAMTS13 antigen levels (iii) a circulating but unidentified inhibitor of human ADAMTS13 activity. Further studies will be required in order to determine the relative importance of each of these three components, and to characterize the molecular mechanisms responsible for ADAMTS13 inhibition. Although we have demonstrated that ULVWF and ADAMTS13 deficiency are both associated with CM and SM, it remains unclear whether these abnormalities constitute epiphenomena, or whether they play active direct roles in mediating the pathophysiology of the condition. However, it is well established that abnormal ULVWF multimers are also present in the circulation in patients with thrombotic thrombocytopenic purpura (TTP) [23],[36]. This rare life-threatening condition is characterized by the development of pathological platelet-rich thrombi in the microvasculature, which in turn results in end-organ dysfunction, principally involving the brain and kidneys. Although inherited or acquired deficiencies of ADAMTS13 have been implicated in the pathogenesis of many cases of TTP [36],[42], recent evidence suggests that ADAMTS13 deficiency is not by itself sufficient to trigger acute TTP. In particular, ADAMTS13 −/− mice are viable, exhibit normal survival, and only develop TTP-like symptoms after specific additional insults (e.g. shigatoxin challenge). Nevertheless, in view of the critical role played by VWF in mediating platelet adhesion/aggregation, and the accumulating evidence suggesting that platelet adhesion/aggregation also facilitate cytoadhesion of IE [43], it seems entirely plausible that ULVWF multimers may indeed be involved in mediating the pathophysiology of severe *P. falciparum* malaria.
